# Exploring the Use of Multiple Mental Health Apps Within a Platform: Secondary Analysis of the IntelliCare Field Trial

**DOI:** 10.2196/11572

**Published:** 2019-03-21

**Authors:** Mary J Kwasny, Stephen M Schueller, Emily Lattie, Elizabeth L Gray, David C Mohr

**Affiliations:** 1 Division of Biostatistics Department of Preventive Medicine Northwestern University Chicago, IL United States; 2 Center for Behavioral Intervention Technologies Department of Preventive Medicine Northwestern University Chicago, IL United States; 3 Department of Psychology and Social Behavior University of California Irvine, CA United States

**Keywords:** mobile apps, depression, anxiety, mobile phone

## Abstract

**Background:**

IntelliCare is a mental health app platform with 14 apps that are *elemental*, *simple and brief* to use, and *eclectic*. Although a variety of apps may improve engagement, leading to better outcomes, they may require navigation aids such as recommender systems that can quickly direct a person to a useful app.

**Objective:**

As the first step toward developing navigation and recommender tools, this study explored app-use patterns across the IntelliCare platform and their relationship with depression and anxiety outcomes.

**Methods:**

This is a secondary analysis of the IntelliCare Field Trial, which recruited people with depression or anxiety. Participants of the trial received 8 weeks of coaching, primarily by text, and weekly random recommendations for apps. App-use metrics included frequency and lifetime use. Depression and anxiety, measured using the Patient Health Questionnaire-9 and Generalized Anxiety Disorder-7, respectively, were assessed at baseline and end of treatment. Cluster analysis was utilized to determine patterns of app use; ordinal logistic regression models and log-rank tests were used to determine if these use metrics alone, or in combination, predicted improvement or remission in depression or anxiety.

**Results:**

The analysis included 96 people who generally followed recommendations to download and try new apps each week. Apps were clustered into 5 groups: Thinking (apps that targeted or relied on thinking), Calming (relaxation and insomnia), Checklists (apps that used checklists), Activity (behavioral activation and activity), and Other. Both overall frequency of use and lifetime use predicted response for depression and anxiety. The Thinking, Calming, and Checklist clusters were associated with improvement in depression and anxiety, and the Activity cluster was associated with improvement in Anxiety only. However, the use of clusters was less strongly associated with improvement than individual app use.

**Conclusions:**

Participants in the field trial remained engaged with a suite of apps for the full 8 weeks of the trial. App-use patterns did fall into clusters, suggesting that some knowledge about the use of one app may be useful in selecting another app that the person is more likely to use and may help suggest apps based on baseline symptomology and personal preference.

## Introduction

### Background

Depression and anxiety are common mental health problems [1,2] and are among the leading causes of morbidity and disability worldwide [3]. The vast majority of people experiencing these common mental health problems are unable to access treatment due to a variety of actual or perceived barriers including the lack of availability of services, time constraints, transportation problems, and cost [4,5]. A wide variety of Web-based treatments have been developed and shown to be highly effective in the treatment of depression and anxiety, particularly when coupled with some human support to promote adherence and enhance outcomes [6,7]. These programs, leveraging the strengths of computer-accessed Web programs in providing information, have strong psychoeducational components along with some interactional components that function much like worksheets [8].

More recently, mobile apps have been developed and evaluated for the treatment of depression [9,10]. Mobile apps have a number of advantages. Because people keep their phones with them, app-based interventions can fit more seamlessly into the fabric of people’s lives. Smartphones are becoming ubiquitous in developed countries and are increasingly common in developing nations [11].

The design of mobile apps for mental health has posed an interesting challenge compared with in-person and Web-based treatments. There are many potential psychological and behavioral strategies that can be used to target specific concerns associated with mental health problems [12,13]. Psychological treatments should be flexible and adaptive, providing treatment elements that best meet the needs and preferences of the patient [14]. Web-based treatments designed to be delivered via a computer can offer a wide variety of treatment approaches, which sometimes require a few layers of navigation. Often, these programs require longer periods of engagement with psychoeducational material, with recommended access every week or few days. However, this design may not be well suited to mobile apps. People tend to use apps in very short bursts of time, sometimes frequently [15,16]. Thus, popular apps tend to be designed for use through simple interactions with limited navigation. A single app tends to focus on a narrow set of objectives. For example, most people do not use one app for transportation needs. Rather, they often use a variety of apps to manage flights on different airlines, manage train transportation and buses, or map driving routes and check traffic. In short, different apps are used flexibly to meet the changing moment-to-moment needs. Thus, there are a large number of behavioral strategies that may be useful for people with common mental health problems, but mobile apps that are used and useful tend to be narrow in focus and quick to use.

The observation of the incongruity between the large variety of potentially useful psychological strategies and the narrow, efficient design requirements for mobile apps has led our team to question the “app for that disorder” approach that has been common in digital mental health design.

IntelliCare addresses this issue by creating a suite of apps, each of which addresses a single psychological or behavioral strategy rather than attempting to address the full theoretical framework for a mental health problem [17]. Thus, each app is elemental, allowing the user to select which strategies are the most useful to them, consistent with the US Institute of Medicine report recommending that app-based treatments combine therapeutic elements as well as consider how people tend to use apps. Each app is simple to use, most requiring less than a minute per engagement [18]. Most of the apps focus on supporting learning or implementing a skill, and not on psychoeducation, thus helping people with an immediate problem. The IntelliCare suite of apps, which has more than 85,000 downloads from the Google Play Store, generally experienced good engagement. A field trial that provided 8 weeks of coaching showed significant reductions in depression and anxiety symptoms.

As we move from a single app that addresses a disorder to a platform of apps that each supports discrete treatment strategies, the management of those apps poses a new challenge. As a mental health app platform such as IntelliCare includes a growing number of apps, user reliance on the trial-and-error method to select apps will make the platform harder to use in a meaningful way. To address this issue, IntelliCare includes a Hub app, which, if downloaded, can help organize users’ experience by making recommendations about which apps to select. Users who download this Hub app use more apps and use the apps for longer periods of time [17]. Receiving a recommendation from the Hub app to use a specific app increases the likelihood that the app will be downloaded and used [19]. However, it is unclear at this point how to recommend these apps in a way that ensures users are presented with options that are likely to meet their preferences and needs.

### Objective

The aim of this study was to investigate the patterns of app use across the IntelliCare platform over time. Specifically, we characterized the general platform use, specific apps use, and use of multiple apps (ie, clusters). We then examined the relationship between general, app, and cluster use and depression or anxiety symptom improvement. Lastly, we explored if there were optimal patterns of general, specific, or cluster of app use in terms of predicting improvement in depression or anxiety symptoms. This study aimed at not only understanding the possible utility of apps but also examining whether outcomes are driven more by user engagement around a construct (or app cluster) or simply by individual app use. Understanding these use patterns could improve our ability to recommend apps that users may engage with or that will be more helpful to them personally.

## Methods

### Participants

This is a secondary analysis of a single-arm field trial of IntelliCare. Details on the methods are reported in the primary paper [18] (trial registration: Clinicaltrials.gov NCT02176226). Briefly, participants were recruited from a variety of sources, including online, a health care system, community advertising, and research registries. Participants were included in this single-arm field trial if they exhibited depressive symptoms indicated by a score of 10 or higher on the Patient Health Questionnaire-9 (PHQ-9) [20] or anxiety symptoms indicated by a score of 8 or higher on the Generalized Anxiety Disorder-7 (GAD-7) questionnaire [21]; were 18 years of age or older (19 years if in Nebraska, given the age of consent); could speak and read English and lived in the United States; and owned and were familiar with using an Android smartphone with data and text plans. Participants were excluded if they had any visual, hearing, voice, or motor impairments that would prevent completion of study procedures; reported having a severe psychiatric disorder (eg, bipolar disorder, psychotic disorder, or dissociative disorder) or any other diagnosis for which this trial was either inappropriate or dangerous; exhibited severe suicidality including a plan and intent; had initiated or changed antidepressant or antianxiolytic pharmacotherapy in the previous 14 days; or had used any of the IntelliCare apps for more than 1 week in the past 3 months. Participants could earn up to US $90 for completion of assessments but were not paid for using the apps or engaging with the coach.

### Procedures

People who met the inclusion criteria based on online questionnaires and signed an online consent approved by the Northwestern University institutional review board were offered 8 weeks of coaching aimed at helping them use the IntelliCare app suite, detailed in the IntelliCare section below. Outcome assessments, which included the PHQ-9 and GAD-7, were administered at baseline and weeks 4 and 8.

### IntelliCare

At the time of this trial, the IntelliCare platform consisted of 14 apps (Multimedia Appendix 1). This included 13 clinical apps, each of which was designed to target a specific behavioral or psychological treatment strategy (eg, goal setting, behavioral activation, and social support) and improve symptoms of depression and anxiety through efficacious treatment strategies [17]. The user’s experience with the clinical apps was coordinated through a Hub app that, among other functions, made weekly recommendations for new apps. Recommendations were made at random, as there was no basis at the time of this trial to select specific apps. Although users were asked to at least try the newly recommended apps, they were encouraged to use the apps they found most helpful.

### Coaching Protocol

Coaching was guided by the IntelliCare Coaching Manual [22], which is based on aspects of the Efficiency Model of Behavioral Intervention Technologies Support [23] and supportive accountability [24]. Coaching was aimed primarily at encouraging participants to try the apps recommended to them through the Hub app. Coaches also answered questions about how to use the tools found in the apps and the rationale behind the skills taught by the apps, encouraged application of the skills in daily life, and provided some technical support as needed. Coaching began with an initial 30- to 45-minute engagement phone call to establish goals for mood and anxiety management, ensure the participant could download the Hub app from the Google Play store, introduce the suite of available smartphone apps, build rapport, and set expectations for the coach-participant relationship. Some participants also received an additional 10-minute call around midtreatment. After the initial engagement call, participants received 2-3 text messages per week from their coach to provide support in using apps, offer encouragement, reinforce app use, and check in on users’ progress or challenges. Coaches also responded to all participant-initiated text messages within 1 working day. The coaches had a dashboard that provided information about the IntelliCare apps on each participant’s phone, including which apps were installed, when they were downloaded, each time an app was used, and which apps were selected as “primary” in the Hub app. The dashboard also included a short message service tool, a section for brief notes, and an alert indicating when no IntelliCare app had been used for 3 days, prompting coaches to check in. Coaches had at least a bachelor’s degree in psychology or a related field and were trained and monitored by one of the authors of the coaching manual.

### App-Use Metrics

Although apps were available for download at any time during the trial, app recommendations were made randomly over the course of 8 weeks, and the time that any individual app was on a participant’s phone could vary substantially. Therefore, in addition to total apps used, other use metrics needed to be defined in relation to the time that the app was on a phone and available to the participant. Accordingly, we created two metrics: *frequency of use* and *lifetime use*. *Frequency of use* was measured as the percentage of days the app was used, calculated as the number of days the app or set of apps was launched divided by the number of days in the study after the app was downloaded by a participant and available for use. For instance, since the trial lasted 8 weeks, or 56 days, if the participant downloaded the app on day 14, the possible number of days it could have been used was 56 minus 14, or 42 days; if they launched the app on 21 of those days, the *frequency of use* would be 50%. We made an *a priori* decision to measure app-use frequency based on the number of days of use rather than the number of times an app was launched, as we were concerned about the numerous potential sources of variability for app use within a day, such as interruptions, or differences in app design that might encourage differential use. *Lifetime use* was defined as the time between the first launch and the last launch. However, we recognized that not every app was designed to be used every day, so we allowed for censoring if the app was still being used in the last week of the trial. For instance, if the app was first launched on day 14 and last launched on day 22, the *lifetime use* of the app would be 8 days. However, if the app was first launched on day 14 and last used in the last week of the study (between days 49 and 56), the *lifetime use* would be censored at 56 minus 14, or 42 days.

### Outcome Assessment

Depression was measured using the PHQ-9 [20] and anxiety was measured using the GAD-7 [25], two commonly used self-report measures. Outcomes were categorized using standard cutoffs [26,27]. For depressed patients, we defined remission as PHQ-9 scores < 5 (typically indicating minimal depression), improvement as 5 ≤ PHQ-9 < 10 (indicating mild-moderate depression), and no improvement as PHQ-9 score ≥ 10 (moderately severe-severe depression). For participants with anxiety, remission was determined as GAD-7 score < 5 (minimal anxiety), improvement as 5 ≤ GAD-7 < 8 (mild anxiety), and no improvement as GAD-7 score ≥ 8 (moderate or severe anxiety).

### Statistical Analyses

We describe app use using visual informatics and descriptive statistics. Additionally, we performed a cluster analysis on the total number of launches by app using a centroid approach with the Spearman correlation to determine if the use of any of the apps was clustered together.

Analyses relating app use to outcome were conducted separately for participants who met the PHQ-9 score ≥10 entry criterion for depression and participants who met the GAD-7 score ≥ 8 criterion for anxiety. We fit ordinal logistic regression models, modeling proportional odds of improvement or remission (I/R) to determine if frequency of use, for any apps combined, individual apps, and clusters of apps varied by outcome. To assess if the use of multiple apps within a cluster was more effective in treating depression or anxiety, we “scored” apps if they had 25% or more frequency of use and then examined if there was a trend between the number of scored apps within a cluster and outcome. Lastly, we employed stepwise selection to determine which apps or combination of apps was most predictive in a model on our ordinal outcomes concerning the I/R of symptoms. All odds ratios (ORs) and 95% CIs are presented for a 10% increase in the number of days used. To examine lifetime use by outcome, we fit Kaplan-Meier plots and used log-rank tests.

All analyses were performed using SASv9.4 (Cary, NC); graphs were created in SASv9.4 or Rv3.4.3 [28]. The type I error was set at .05 for all analyses, but we caution that any findings should be further investigated, as this is a secondary analysis and therefore subject to increased type I errors.

## Results

### Participants

A detailed description of the participants and primary outcomes has been published elsewhere [18]. Briefly, 99 participants were enrolled and began the 8-week field trial. A flow diagram is available in the main outcome paper [18]; only 3 participants were lost to follow-up, leaving 96 participants with at least two outcome assessments, who are the focus of this secondary analysis. Among those participants, the median age was 36 (interquartile range: 27-52) years, 74 (77%) were women, 80 (84%) were non-Hispanic white individuals, 60 (63%) were on medication for anxiety or depression, and 24 (25%) were currently receiving psychotherapy. At the start of the field trial, the depression criterion of PHQ-9 score ≥10 was met by 78 (81%) participants, the anxiety criterion of GAD-7 score ≥ 8 was met by 77 (80%) participants, and the criteria for both depression and anxiety were met by 59 (61%) participants. Using our classification of treatment response, among the 78 participants meeting the entry criterion for depression, by the end of treatment, 26 (33%) were in remission, 30 (38%) had improved symptoms, and 22 (28%) remained symptomatic. Of the 77 participants who met the criteria for anxiety, 28 (36%) were in remission, 23 (30%) had improved symptoms, and 26 (34%) remained symptomatic.

### General App Use

**Figure 1:**
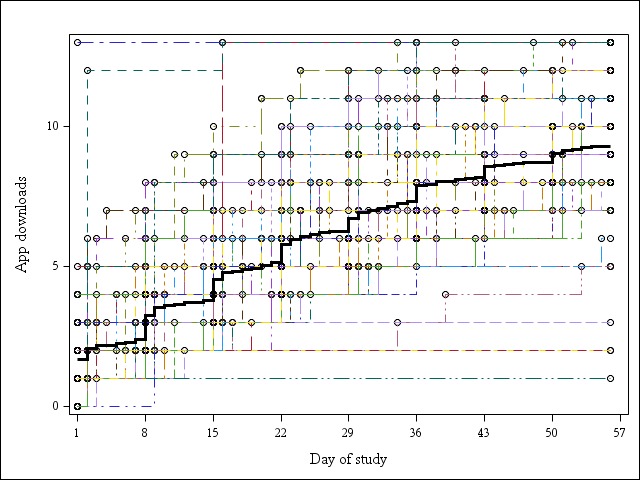
Cumulative downloads of apps by study day.

Participants downloaded an average of 9.3 apps (SD 2.6) over 8 weeks; half of the participants used over 9 apps. Overall, participants tended to download the apps in the suite gradually over the 8 weeks, rather than download all the apps at the start of the trial. There was an immediate launch of a few apps in the first week (average number downloaded 2.4; SD 2.0), which tapered gradually over the length of the study, with average increases of 1.4 apps/week for weeks 2 and 3, and roughly 1 app/week for weeks 4-6, down to 0.5 apps/week for weeks 7 and 8 (Figure 1). The apps were used throughout the trial, with a median lifetime use of 55 (interquartile range: 53-55) days for the suite.

### Individual App Use

**Figure 2:**
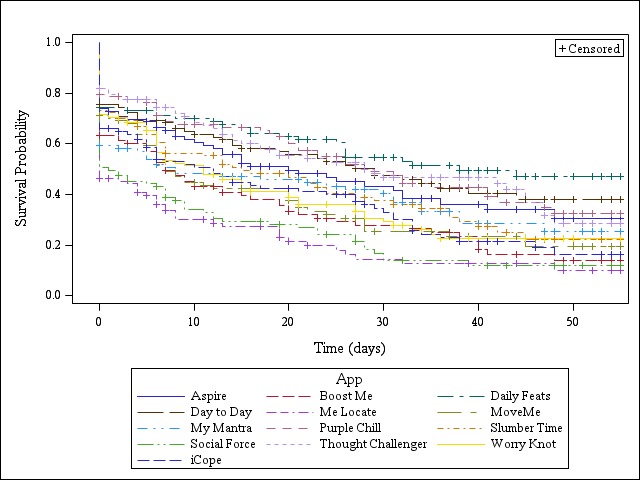
Lifetime use of each individual app.

Half of the participants used at least one IntelliCare treatment app for 49 (88%) of the 56 days, with an interquartile range of 35 (63%) to 53 (95%) days. Median percent of days used for specific apps ranged from 2% (Me Locate) to 41% (Daily Feats) (Table 1). The lifetime use for specific apps ranged from 0 days (Me Locate) to 21 days (Daily Feats). Kaplan-Meier plots for each app’s lifetime use over the trial period are shown in Figure 2.

**Table 1:** Usage of all Intellicare apps.

Usage pattern and apps		Overall use		Use by participants who downloaded the app		
		Frequency of Use, median (25th, 75th)	Lifetime Use^a^, median (25th, 75th)	Number downloaded (%)	Frequency of Use, median (25th, 75th)	Lifetime Use^a^, median (25th, 75th)
**Individual app use**						
	Aspire	19 (3, 50)	13 (0, 32)	73 (76)	30 (16, 62)	20 (8, 35)
	Boost Me	12 (0, 25)	7 (0, 22)	65 (68)	19 (12, 33)	13 (6, 33)
	Daily Feats	41 (1, 75)	21 (0, 39)	72 (75)	54 (33, 88)	27 (16, 45)
	iCope	11 (0, 25)	6 (0, 30)	69 (72)	17 (10, 42)	20 (5, 33)
	My Mantra	16 (0, 58)	6 (0, 30)	59 (61)	43 (18, 78)	25 (7, 37)
	Me Locate	2 (0, 17)	0 (0, 8)	50 (52)	17 (12, 31)	8 (4, 20)
	Day to day	26 (4, 57)	16 (0, 36)	76 (79)	41 (21, 67)	25 (9, 41)
	MoveMe	16 (2, 33)	6 (0, 23)	73 (76)	21 (12, 38)	11 (5, 27)
	Purple Chill	24 (5, 62)	19 (3, 40)	79 (82)	34 (17, 68)	23 (8, 41)
	Slumber Time	25 (0, 57)	13 (0, 34)	71 (74)	41 (22, 67)	22 (7, 37)
	Social Force	5 (0, 22)	1 (0, 15)	54 (56)	17 (9, 35)	12 (6, 27)
	Thought Challenger	21 (5, 56)	17 (4, 37)	80 (83)	30 (14, 65)	21 (10, 42)
	Worry Knot	13 (1, 29)	7 (0, 28)	72 (75)	19 (12, 46)	15 (6, 32)
**Cluster use**						
	Thinking	54 (30, 83)	45 (29, 55)	93 (97)	55 (32, 83)	46 (33, 55)
	Calming	45 (20, 66)	34 (13, 48)	87 (91)	48 (26, 71)	37 (19, 49)
	Checklists	48 (26, 83)	37 (16, 49)	84 (88%)	52 (34, 86)	41 (25, 50)
	Activity	25 (12, 43)	28 (7, 41)	83 (86%)	29 (14, 47)	33 (13, 44)
	Other	15 (2, 32)	6 (0, 23)	73 (76%)	21 (12, 37)	11 (5, 27)

^a^Lifetime Use is defined as the time between the first launch and the last launch.

### Clusters of Apps

A cluster analysis revealed 5 groups of apps that tended to be used together. The average within-cluster R^2^ ranged from .34 to .61, whereas the average R^2^ to the nearest cluster was .03 to .33. In total, the 5 clusters explained 59% of the variability of correlations between launches. The five clusters identified could best be described as follows: “Thinking” - Thought Challenger, MyMantra, Day to Day, and iCope; “Calming” - Purple Chill and Slumber Time; “Checklists” - Aspire and Daily Feats; “Activity” - Boost Me and MoveMe; and “Other” - Me Locate, Social Force, and Worry Knot, which appeared unified only in the lack of engagement relative to other apps.

The Thinking and Checklist clusters were used most often and for a longer period of time than the other clusters, with median lifetimes over 5 weeks in the 8-week trial. The Calming and Activity clusters were also used fairly often, with a median use of just over 4 weeks. The Other cluster was used the least, with a median lifetime of a week (Table 1). The number of apps scored (≥25% frequency of use) in each cluster was fairly diverse for the Thinking, Calming, and Checklist clusters, where there were participants who did not use any of the apps much as well as participants who used all the apps within the cluster. The Activity and the Other clusters were more prone to have individuals who did not use any of the apps often (Table 2).

**Table 2:** Number of apps with more than 25% frequency of use within each app cluster.

Number of apps with ≥25% use	Thinking, n (%)	Calming, n (%)	Checklist, n (%)	Activity, n (%)	Other, n (%)
0	25 (26)	29 (30)	23 (24)	52 (54)	49 (51)
1	21 (22)	36 (38)	40 (42)	32 (33)	31 (32)
2	22 (23)	31 (32)	33 (34)	12 (13)	12 (13)
3	17 (18)	N/A^a^	N/A	N/A	4 (4)
4	11 (11)	N/A	N/A	N/A	N/A

^a^N/A: not applicable.

**Table 3:** Ordinal odds ratios for improvement or remission of depression/anxiety by app use.

Item		Depression	Anxiety
**Univariate odds ratio (95% CI) for suite**			
	Number of apps downloaded	1.07 (0.91-1.25)	1.20 (1.01-1.43)
	Percent days of app use	1.26 (1.05-1.52)	1.19 (0.99-1.43)
**Univariate odds ratio (95% CI) for individual apps**			
	Aspire	1.15 (1.00-1.32)	1.19 (0.99-1.43)
	Boost Me	1.27 (1.00-1.61)	1.37 (1.06-1.77)
	Daily Feats	1.09 (0.97-1.22)	1.05 (0.94-1.18)
	Day to day	1.11 (0.98-1.27)	1.18 (1.03-1.35)
	iCope	1.00 (0.86-1.16)	1.01 (0.86-1.17)
	My Mantra	1.02 (0.91-1.15)	1.13 (0.99-1.28)
	Me Locate	1.15 (0.93-1.41)	1.82 (1.25-2.63)
	MoveMe	1.15 (0.99-1.34)	1.17 (0.99-1.37)
	Purple Chill	1.17 (1.02-1.34)	1.16 (1.01-1.34)
	Slumber Time	1.04 (0.92-1.18)	1.07 (0.94-1.23)
	Social Force	1.05 (0.89-1.25)	1.16 (0.96-1.42)
	Thought Challenger	1.07 (0.93-1.22)	1.15 (1.01-1.31)
	Worry Knot	1.04 (0.90-1.21)	1.06 (0.91-1.23)
**Univariate odds ratio (95% CI) for clusters**			
	Thinking	1.16 (1.01-1.33)	1.17 (1.02-1.35)
	Calming	1.17 (1.02-1.35)	1.18 (1.02-1.36)
	Checklist	1.18 (1.04-1.34)	1.14 (1.00-1.30)
	Activity	1.08 (0.92-1.27)	1.26 (1.05-1.52)
	Other	1.17 (0.99-1.37)	1.16 (0.99-1.36)
**Multivariate models**			
	Boost Me	1.31 (1.02-1.67)	—^b^
	Lifetime Purple Chill^a^	1.25 (1.07-1.47)	—
	Me Locate	—	1.74 (1.19-2.55)
	Lifetime Thought Challenger^a^	—	1.20 (1.02-1.43)

^a^As lifetime is measured in days, and 1 extra day of use is clinically meaningless, odds ratios are based on a unit difference of 1 week.

^b^Not available.

### Relationship Between Suite Use and Outcome

The total number of apps used was not associated with I/R in depression (OR=1.07; 95% CI=0.91-1.25), but was associated with I/R in anxiety (OR=1.20; 95% CI=1.01-1.43). Conversely, the frequency of use of any app use was associated with the odds of I/R of depression (OR=1.26; 95% CI=1.05-1.52), but it was not associated with I/R of anxiety (OR=1.19; 95% CI=0.99-1.43) (Table 3).

### Relationship Between App Use and Outcome

For depression outcomes, frequency of use of Aspire was associated with increased odds of I/R (OR=1.15; 95% CI=1.00-1.32), and the frequency of use of Purple Chill was associated with increased odds of I/R, but the assumption of proportional odds was not met and increased odds of remission was seen only among those who showed improvement (OR=1.39; 95% CI=1.11- 1.72). For anxiety outcomes, frequency of use of Boost Me (OR=1.37; 95% CI=1.06-1.77), Day to Day (OR=1.82; 95% CI=103-1.35), Me Locate (OR=1.82; 95% CI=1.25-2.63), Purple Chill (OR=1.16; 95% CI=1.01-1.34), and Thought Challenger (OR=1.15; 95% CI=1.01-1.31) were associated with increased odds of I/R (Table 3).

**Figure 3:**
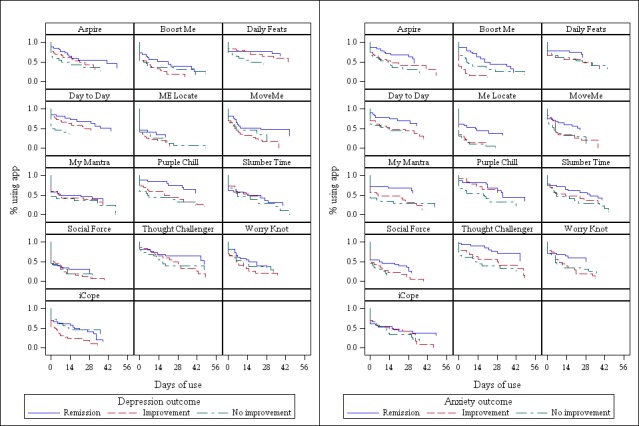
Lifetime use of individual apps by depression and anxiety outcomes.

There were significant differences in the lifetime use for iCope, MoveMe, and Purple Chill between participants who had remission, improvement, or no improvement for depression (Log-rank *P*=.02, .04, and .02, respectively) (Figure 3). Among those with anxiety, lifetime use of Boost Me, My Mantra, Me Locate, MoveMe, Thought Challenger, and Worry Knot varied across remission, improvement, and no improvement statuses (*P=*.001, .02, .001, .04, .008, and .04, respectively) (Figure 3).

### Relationship Between Cluster Use and Outcome

Among participants entering the study with high levels of depressive symptoms, the odds of I/R were significantly greater for participants with a higher frequency of use for apps in the Thinking cluster (OR=1.16; 95% CI=1.01-1.33), the Calming cluster (OR=1.17; 95% CI=1.02-1.35), and the Checklist cluster (OR=1.18; 95% CI=1.04-1.34) (Table 3). We also examined whether the number of apps used within a cluster was related to outcome. There was a significant trend in I/R when multiple apps were used in the Checklist cluster (OR=1.96; 95% CI=1.12-3.44) as well as the Activity cluster (OR=1.81; 95% CI=1.00-3.26). However, in stepwise multivariable ordinal regression models, after adjusting for frequency of use in the Checklist cluster, no other cluster use or number of apps used within a cluster increased the odds of I/R.

Among participants with high levels of anxiety symptoms, the odds of I/R were significantly greater for those with a higher frequency of use in the Thinking cluster (OR=1.17; 95% CI=1.02-1.35), the Calming cluster (OR=1.18; 95% CI=1.02-1.36), the Checklist cluster (OR=1.14; 95% CI=1.00-1.30), and the Activity cluster (OR=1.26; 95% CI=1.05-1.52) (Table 3). Additionally, there was a significant trend in I/R, as the number of apps used increased within the Thinking cluster (OR=1.47; 95% CI=1.07-2.02)), the Checklist cluster (OR=1.79; 95% CI=1.03-3.13), the Activity cluster (OR=1.87; 95% CI=1.03-3.39), and the Other cluster (OR=2.19; 95% CI=1.24-3.89). After adjusting for frequency of use in the Activity cluster, no other cluster or number of apps used within a cluster increased the odds of I/R in multivariable ordinal regression models.

**Figure 4:**
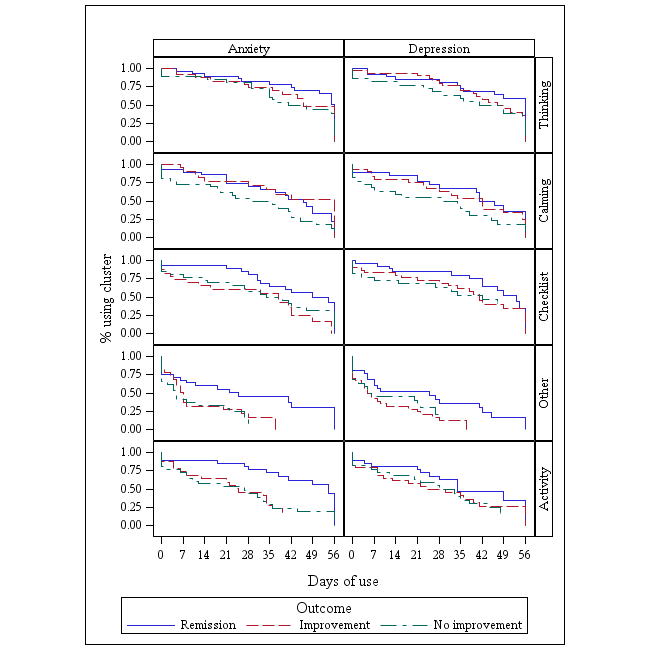
Lifetime use of each cluster of apps by anxiety and depression outcomes.

For depression, longer lifetime use of the Activity cluster was significantly associated with better outcomes (Log-Rank *P*=.02). For anxiety, longer use of the Calming, Activity, and Other clusters was associated with better outcomes (Log-Rank *P*=.01, .03, and .002, respectively) (Figure 4).

### Optimal Use Patterns

For participants with depression, the frequency of use of Boost Me in combination with lifetime use of Purple Chill was the most predictive combination for response. After adjusting for the frequency of use of Boost Me and the lifetime use of Purple Chill, no other app or cluster use metric was significantly associated with I/R. For participants with anxiety, the frequency of use of Me Locate and lifetime use of Thought Challenger were the most predictive use combination of apps for I/R. After adjusting for these, no other app or cluster use metric was significantly associated with I/R (Table 3).

## Discussion

### Principal Results

Determining the definition of “use” for apps can be challenging. Here, we defined two metrics: *frequency* *of use* (percent of days used) and *lifetime use* (time between initial and last launch). Additionally, we examined clusters of app use based on correlations between the total number of launches and were able to identify groups of apps that could be defined based on behavioral strategy and user interaction style. This revealed five clusters. The Thinking cluster included apps that prompt or rely on a person to use cognitive processes. The Calming cluster provided tools for relaxation and strategies to improve sleep. The Checklist cluster was defined by the type of interaction people had with the app—the use of checklists—rather than by a psychological strategy. This underscores that the design and interaction features used in apps may be as important to people’s preferences as the psychological goal or behavioral strategies. The Activity cluster was defined by apps that targeted behavioral activation and physical activity. A fifth cluster, which we called Other, consisted of apps that may need further development. Two of the three had the lowest use, and the third—Worry Knot—had an interaction design that was often not well received, based on user feedback. Nonetheless, the fact that the clusters based on use were well defined suggests that recommendation systems could be useful in getting apps to people that are more likely to be used.

We explored the relationship between individual apps and outcome. For depression, Purple Chill (relaxation) and Aspire (living one’s values) were predictive of improvement, while Boost Me (behavioral activation), Day to Day (psychoeducation), Me Locate (used geofencing), Purple Chill, and Thought Challenger (cognitive restructuring) were associated with improvement in anxiety. Given that app use was in the context of a suite, it is difficult to interpret these findings. It is likely that improvement is not necessarily due to the use of “an app,” but rather a mix of apps.

One hypothesis we proposed was that targeting a construct (eg, Thinking or Calming) through use of a set of apps may be more beneficial than the use of any individual app. Indeed, the Thinking, Calming, and Checklist clusters were all related to improvement in depression, and those three clusters, along with Activity, were associated with improvement in anxiety. However, our stepwise models did not conform to this hypothesis. When individual apps and clusters were analyzed together, the frequency of Boost Me and lifetime use of Purple Chill were associated with improvement in depression, while the frequency of Me Locate and lifetime use of Thought Challenger were associated with improvement in anxiety. Thus, our hypothesis regarding the use of clusters for improvement received partial support, but was not robust in the presence of all app-use data.

Finally, we note that engagement remained high throughout the study. We have noted previously that in the public deployment of IntelliCare through the Google Play Store, providing recommendations, even randomly, as was done in the field trial, increases the likelihood that an individual will download an app [19]. However, it was unclear how people would use apps in the context of a treatment where all apps were available from the start. In this study, we see that people tended not to download all the apps at once, but rather wait for the weekly recommendations to download and initiate use.

This study provides the first view of how digital mental health platforms that provide a variety of apps or treatments may be optimized. These findings suggest that some knowledge about a person’s use of one app may be helpful in selecting the next app to recommend. There is some support for the idea that use of clusters may also be helpful in improving symptoms, although these findings were not robust. Together, these findings support the idea that recommendation engines may be useful in promoting use in platforms with multiple apps such as IntelliCare and promoting symptom improvement. This will be critical since, to maintain engagement with an app platform, it will be important to quickly connect people to apps that they want to use. For instance, a recommender system would be helpful in that someone with depression might have Purple Chill and Boost Me recommended first. If the person did not engage with those apps, perhaps other apps in similar clusters could be recommended, like Slumber Time or MoveMe. In a broader perspective, a clinician might recommend apps focused on relaxation or apps geared towards living one’s values for depressed patients.

### Limitations

There are limitations to this study that should be considered when interpreting these findings. Chief among them is that we have performed a large number of analyses for the sample size. Thus, some of findings may be spurious, and in other cases, we were likely underpowered for the number of variables included in analyses. Additionally, the sample size was restrictive; therefore, we could not account for baseline demographics which may also cluster with app use.

As in any secondary analysis, data obtained were from a field trial that restricted participation in order to assess the effectiveness of the apps in changing symptoms of depression and anxiety. Our findings may reasonably apply to English-speaking adults living in the United States, owing to ownership of an internet-ready Android mobile phone with data and text plans and without any visual, hearing, voice, or motor impairments; severe psychiatric disorders; or suicidality exhibition.

### Conclusions

We found that a suite of apps was engaging to participants in a field trial for treatment of depression or anxiety. Despite all apps being available for immediate download, participants gradually downloaded and engaged with various apps throughout the trial. App-use patterns fell into clusters, suggesting that some knowledge about the use of one app may be useful in helping select another app that the person is more likely to use. This could provide the basis for making more targeted recommendations based on app-use data.

Although the use metrics of different apps in the suite are correlated, a stepwise analysis showed that the use of Boost Me (an Activity-focused app) and sustained use of Purple Chill (a Calming app) were most effective at improving depression, while the use of Me Locate and sustained use of Thought Challenger (a Thinking app) were most effective at improving anxiety.

## Appendix

Multimedia Appendix 1 Description of the IntelliCare apps.

## Abbreviations

GAD-7: Generalized Anxiety Disorder-7
I/R: improvement or remission
N/A: not applicable
OR: odds ratio
PHQ-9: Patient Health Questionnaire-9
