# Supplementary material for: Exploring the Use of Multiple Mental Health Apps Within a Platform: Secondary Analysis of the IntelliCare Field Trial
Source: JMIR Ment Health. 2019 Mar 21;6(3):e11572. doi: 10.2196/11572 (PMC6447993; doi:10.2196/11572)
Supplement: Multimedia Appendix 1 [file mental_v6i3e11572_app1.pdf]

Appendix Table 1: Description of Intellicare Apps[a]

App	Behavioral strategy	Description
IntelliCare Hub		Manages messages and notifications from the other apps within the IntelliCare collection.
Aspire	Personal values and goal setting	Guides user to identify the values that guide one’s life and the actions (or “paths”) that one does to live that value. Helps keep track of those actions throughout the day and supports the user in living a more purpose-driven and satisfying life.
Day to Day	Psychoeducation and prompts	Delivers a daily stream of tips, tricks, and other information throughout the day to boost the user’s mood. Prompts the user to work on a particular theme each day and every week; learn more about how to effectively cultivate gratitude, activate pleasure, increase connectedness, solve problems, and challenge one’s thinking.
Daily Feats	Goal setting	Encourages the user to incorporate worthwhile and productive activities into the day. Users add accomplishments to the Feats calendar, where they can track their positive activity streaks and level up by completing more tasks. Helps motivate users to spend their days in more meaningful, rewarding ways to increase overall satisfaction in life.
Worry Knot	Emotional regulation and exposure	Teaches the user to manage worry with lessons, distractions, and a worry management tool. Provides a guided tool to address specific problems that a user cannot stop thinking about and provides written text about how to cope with “tangled thinking.” Presents statistics about progress as the user practices coping with worry, gives daily tips and tricks about managing worry, and provides customizable suggestions for ways to distract oneself.
ME Locate	Behavioral activation	Provides a personal map for finding and saving user’s mood-boosting locations. Assists the user in finding and remembering these places to help them make plans, maintain a positive mood, and stay on top of responsibilities.
Social Force	Social support	Prompts the user to identify supportive people in their lives, and provides encouragement for the user to get back in touch with those positive people.

App	Behavioral strategy	Description
My Mantra	Self-affirmations and positive reminiscence	Prompts the user to create mantras (or repeatable phrases that highlight personal photo strengths and values and can motivate one to do and feel good) and construct virtual albums to serve as encouragement and reminders of these mantras.
Thought Challenger	Cognitive reframing	Guides the user through an interactive cognitive restructuring tool to examine thoughts that might exaggerate negative experiences, lead one to be overcritical, and bring down one's mood. Teaches the user to get into the habit of changing perspective and moving toward a more balanced outlook on life.
iCope	Proactive coping	Allows the user to send oneself inspirational messages and reassuring statements, written in their own words, to help the user get through tough spots or challenging situations.
Purple Chill	Relaxation	Provides users with a library of audio recordings to relax and unwind. Teaches a variety of relaxation and mindfulness practices to destress and worry less.
MoveMe	Exercise for mood	Helps the user select exercises to improve mood. Provides access to curated exercise videos and to written lessons about staying motivated to exercise. Allows the user to schedule motivational exercise time for oneself throughout the week.
Slumber Time	Sleep hygiene	Prompts the user to complete sleep diaries to track sleep. Provides a bedtime checklist intended to clear one's mind before going to sleep. Provides audio recordings to facilitate rest and relaxation. Features an alarm clock function.
Boost Me	Behavioral activation	Encourages users to select and schedule positive activities ("boosts") when they notice a drop in mood and to track positive activities they note positively impacting their mood. Includes animated mood tracking for pre or post positive activities, calendar integration, and suggested activities that are auto-populated based on past mood improvement.

[a]Printed previously: Mohr DC, Noth Tomasino K, Lattie EG, Palac HL, Kwasny MJ, Weingardt K, Karr CJ, Kaiser SM, Rossom RC, Bardsley LR, Caccamo L, Stiles-Shields C, Schueller SM. IntelliCare: An Eclectic, Skills-Based App Suite for the Treatment of Depression and Anxiety. J Med Internet Res 2017;19(1):e10
